# Emergence of Fatal PRRSV Variants: Unparalleled Outbreaks of Atypical PRRS in China and Molecular Dissection of the Unique Hallmark

**DOI:** 10.1371/journal.pone.0000526

**Published:** 2007-06-13

**Authors:** Kegong Tian, Xiuling Yu, Tiezhu Zhao, Youjun Feng, Zhen Cao, Chuanbin Wang, Yan Hu, Xizhao Chen, Dongmei Hu, Xinsheng Tian, Di Liu, Shuo Zhang, Xiaoyu Deng, Yinqiao Ding, Lu Yang, Yunxia Zhang, Haixia Xiao, Mingming Qiao, Bin Wang, Lili Hou, Xiaoying Wang, Xinyan Yang, Liping Kang, Ming Sun, Ping Jin, Shujuan Wang, Yoshihiro Kitamura, Jinghua Yan, George F. Gao

**Affiliations:** 1 China Animal Disease Control Center, Beijing, China; 2 Center for Molecular Immunology, Institute of Microbiology, Chinese Academy of Sciences, Beijing, China; 3 Graduate University, Chinese Academy of Sciences, Beijing, China; 4 College of Veterinary Medicine, China Agricultural University, Beijing, China; 5 China-Japan Joint Laboratory of Molecular Immunology and Molecular Microbiology, Institute of Microbiology, Chinese Academy of Sciences, Beijing, China; US Naval Medical Research Center Detachment/Centers for Disease Control, United States of America

## Abstract

Porcine reproductive and respiratory syndrome (PRRS) is a severe viral disease in pigs, causing great economic losses worldwide each year. The causative agent of the disease, PRRS virus (PRRSV), is a member of the family *Arteriviridae*. Here we report our investigation of the unparalleled large-scale outbreaks of an originally unknown, but so-called “high fever” disease in China in 2006 with the essence of PRRS, which spread to more than 10 provinces (autonomous cities or regions) and affected over 2,000,000 pigs with about 400,000 fatal cases. Different from the typical PRRS, numerous adult sows were also infected by the “high fever” disease. This atypical PRRS pandemic was initially identified as a hog cholera-like disease manifesting neurological symptoms (e.g., shivering), high fever (40–42°C), erythematous blanching rash, etc. Autopsies combined with immunological analyses clearly showed that multiple organs were infected by highly pathogenic PRRSVs with severe pathological changes observed. Whole-genome analysis of the isolated viruses revealed that these PRRSV isolates are grouped into Type II and are highly homologous to HB-1, a Chinese strain of PRRSV (96.5% nucleotide identity). More importantly, we observed a unique molecular hallmark in these viral isolates, namely a discontinuous deletion of 30 amino acids in nonstructural protein 2 (NSP2). Taken together, this is the first comprehensive report documenting the 2006 epidemic of atypical PRRS outbreak in China and identifying the 30 amino-acid deletion in NSP2, a novel determining factor for virulence which may be implicated in the high pathogenicity of PRRSV, and will stimulate further study by using the infectious cDNA clone technique.

## Introduction

Porcine reproductive and respiratory syndrome (PRRS) is recognized as a serious swine disease and is characterized with either reproductive failure in pregnant sows, or respiratory tract distress particularly in sucking pigs [1]–[3]. This viral disease was first discovered in the United States in 1987 [4]–[6], subsequently found in Europe [4], and identified in Asia in the early 1990s [7], [8]. To date, PRRS has spread worldwide with the characteristics of endemic in those swine-cultivating countries, causing enormous economic losses each year [4], [9]–[12]. The etiological agent of PRRS is porcine reproductive and respiratory syndrome virus (PRRSV) which, together with lactate dehydrogenase-elevating virus of mice (LDEV), equine arteritis virus (EAV), and simian hemorrhagic fever virus (SHFV), belongs to the family *Arteriviridae* within the order *Nidovirales* [10], [13], [14].

PRRSV, a member of the small enveloped viruses, has a single-strand positive-sense RNA (+ssRNA) genome of approximately 15.1–15.5 kb, comprising at least 8 open reading frames (ORFs) that encode about 20 putative proteins [13], [15]. The genome also contains two untranslated regions (UTR) at both the 5′- and 3′-ends [13], [15]. In detail, ORF1 (ORF1a and ORF1b) is located downstream of the 5′-UTR and occupies more than two-thirds of the whole genome. ORF1a is translated directly, whereas ORF1b is translated by a ribosomal frameshift, yielding a large ORF1ab poly-protein that is proteolytically cleaved into products related to the virus transcription and replication machinery [16], [17]. ORFs 2–7, located upstream of the 3′-UTR, encode a series of viral structural proteins associated with the virion, such as the envelope protein (E) and nucleocapsid protein (N) [15], [18], [19]. These proteins are all translated from a 3′-coterminal nested set of sub-genomic mRNAs (sgmRNAs) [1].

Phylogenetic analysis of PRRSV isolates from different geographical regions worldwide clearly indicates the existence of two major genotypes: Type I representing the European prototype (Lelystad virus, LV), and Type II with the Northern American strain ATCC VR2332 as a prototype [15], [20], [21]. Moreover, some studies have shown that ORF5 and the non-structural protein 2 (NSP2)-coding gene (*nsp2*) may represent the most genetically variable regions in PRRSV genomes [16], [18], [22]–[25]. It is also well documented that PRRSV strains differ greatly in their pathogenicities [9], [26]–[28]. Vaccination is currently considered to be an appropriate strategy for prevention of PRRS, although its bio-safety may need further evaluation. Recently, it was suggested that several newly identified virulent PRRSV isolates have been introduced into swine populations through the inoculation of PRRSV-derived inactivated vaccines [29]–[33]. Meanwhile, one vital step towards understanding PRRSV pathogenesis has been achieved by developing the infectious cDNA clones [34]–[38].

In this study, we report, for the first time, the large-scale devastating outbreak of PRRS in China during the summer of 2006. The outbreak, known locally as “high fever”, overwhelmed about 10 provinces (autonomous cities or regions) with more than 2,000,000 cases of pig infection. A systematic investigation, ranging from epidemiology and pathology to comparative genomics, was carried out with the aim of understanding the pathogenesis of this special PRSSV. A novel potent pathogenicity island (PAI) or virulence-associated factor was identified, and animal experiments reproduced the high virulence and the clinically-observed features. Together, our report described the detailed investigation (clinically and experimentally) of this widespread outbreak of PRRS in China and identification of a new PRRSV variant, causing severe disease in pigs including the adults.

## Results

### Epidemiological and Clinical Features

**Figure 1:**
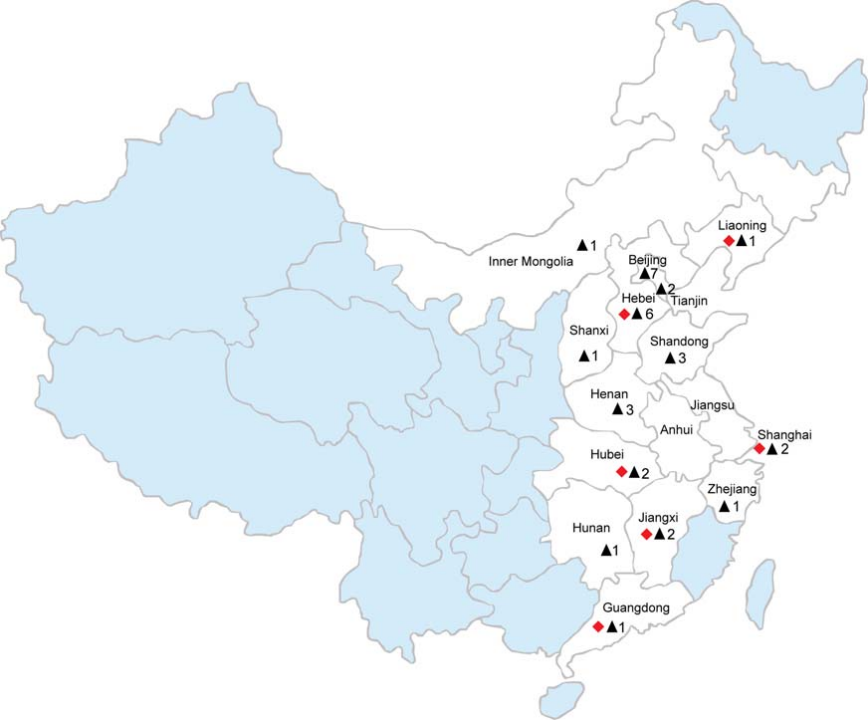
Outbreak sites of the 2006 PRRS epidemic in China. The provinces or autonomous cities (regions) affected are indicated in white. The PRRSV isolates from the endemic sites (highlighted with red diamonds) have been sequenced at the whole genome level, while others from outbreak places (indicated in black triangles) have had selected genes sequenced, such as ORF5, and *nsp2*.

**Figure 2:**
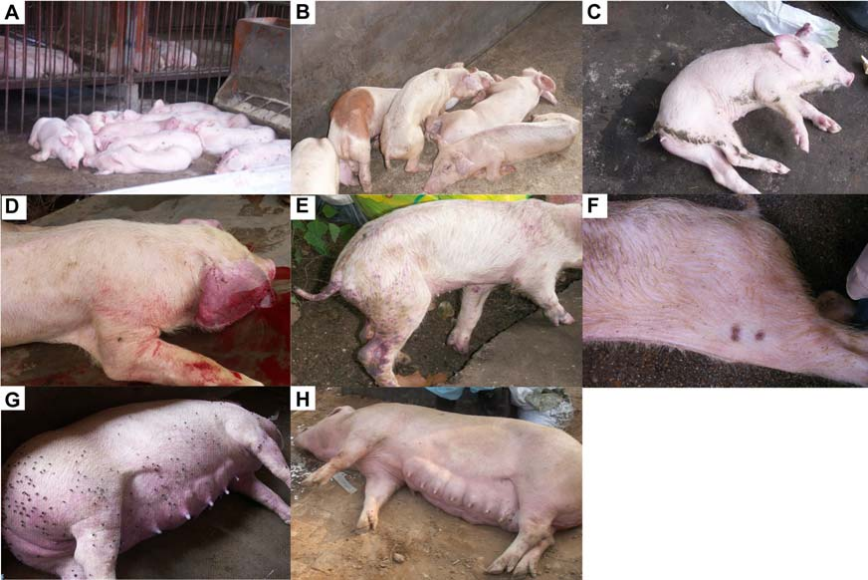
Clinical presentation of pigs with “high fever” disease. (A) Sick pigs with a fat and healthy appearance. (B) Sick pigs with thin and debilitated features. (C) The shivering piglet. (D) The limping pig with erythematous blanching rash in its ears. (E) & (F) Pimples observed on the back of an infected pig. (G) & (H) Grown pigs killed during this epidemic.

In June 2006, a previously unknown severe disease designated “high fever” occurred in several pig farms and subsequently overwhelmed almost half of China. More than 10 provinces (autonomous cities), including Jiangxi Province, Hebei Province, and Shanghai City, were affected by the pandemic (Fig. 1). Initially, the “high fever” was suspected to be hog cholera or African swine fever (ASF). The representative sick pigs had the following common clinical symptoms (Fig. 2): rubefaction, blood spots, petechiae, erythematous blanching rashes, and pimples, frequently observed in ears (Fig. 2D), mouth, noses, back (Fig. 2E&F), and the inner thigh. Other common symptoms included high fever (40–42°C), depression, anorexia, cough, asthma, lameness (Fig. 2A&D), shivering (Fig. 2C), disorder in the respiratory tract, and diarrhea. Infected pigs could be divided into two groups: group one, which appeared fat and healthy (Fig. 2A); and group two with thin and debilitated features (Fig. 2B), ultimately leading to death (Fig. 2). To our surprise, many grown pigs also died during this epidemic period (Fig. 2G&H), which is unlike the case for typical PRRSV infection.

The disease course varied from 5 to 20 days. Infected pigs were highly contagious, usually affecting the whole pig population within 3–5 days. The first pig death case was recorded on the 7^th^ day post-monitoring. The epidemic persisted for several months without any obvious improvements, due partly to poor initial knowledge of the etiological agent as well as the limited efficacy of the routine treatments. Consequently, this large-scale outbreak of so-called “high fever” disease has caused enormous economic losses, especially for pig farmers. According to authoritative statistics from China Animal Disease Control Center (CADC), this epidemic ended in September, affected about 2,120,000 pigs and claimed a death toll of no less than 400,000 pigs, with an average death rate of 19.68% (Table 1).

**Table 1:** Incomplete statistics for the 2006 PRRS epidemic in China

Type	Number (×10,000)																
	Zhe Jiang	He Nan	Hu Nan	Shan Dong	Shang Hai	Hu Bei	Liao Ning	Jiang Xi	Shan Xi	He Bei	Inner Mongolia	Tian Jing	Guang Dong	Bei Jing	An Hui	Jiang Su	Sum
Sick pigs	9.61	/	59.85	/	/	14.41	/	32	/	/	1.1	/	/	/	44.2	51.14	212.31
Fatal pigs	2.74	/	19.20	/	/	2.5	/	6	/	/	0.026	/	/	/	3.84	7.49	41.79
PRRSV	+	+	+	+	+	+	+	+	+	+	+	+	+	+	+	+	

/: uncounted data; +: positive in PRRSV detection by random sampling; the average death rate: 19.68% (41.79/212.31)

### Pathological Characterization of the Infected Pigs

**Figure 3:**
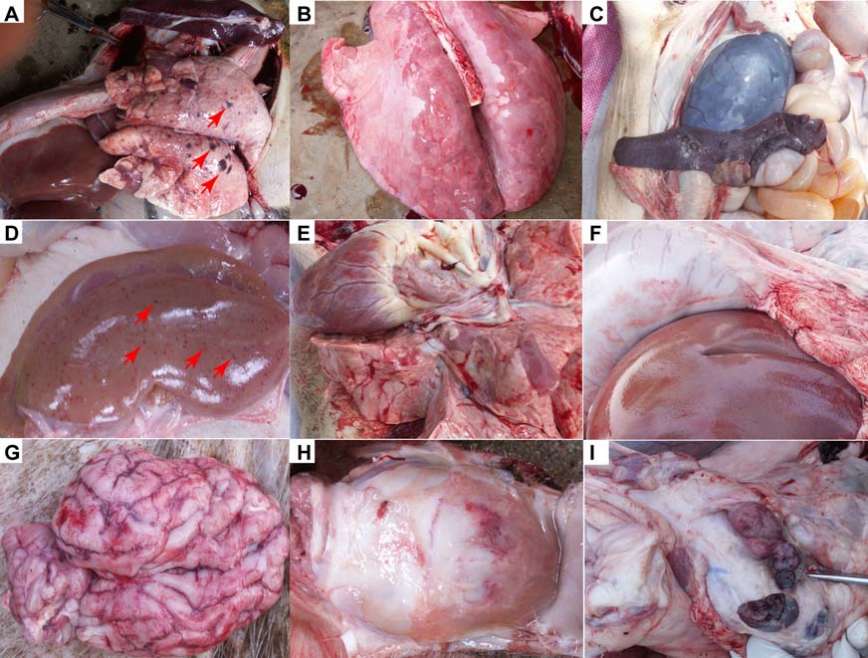
Severe damage to multiple organs in dead pigs. Lung haemorrhage (indicated at the arrow). (B) lung edema. (C) Spleen infarct and bladder dilatation filled with mahogany urine. (D) Kidney with many blood spots (at the arrows). (E) Heart with disorders. (F) Liver with yellow-white necrosis or haemorrhage. (G) Encephala softened slightly. (H) Brain putamen with blood egression emission. (I) Lymph node with haemorrhagic spots

**Figure 4:**
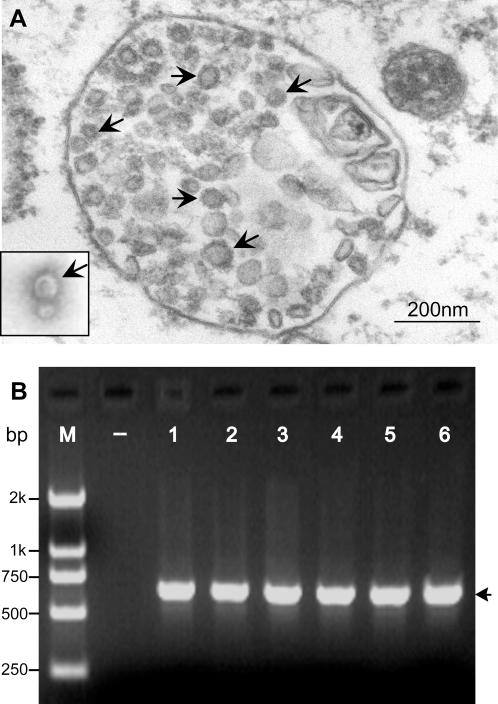
Qualitative detection of the viral pathogen, PRRSV. (A) PRRSV particles under the electron microscopy (EM). The PRRSV particles (indicated by arrows) are visualized in the cytoplasm of Marc-145 cells (in ultra-thin sections), and the PRRSVs are also observed in the TEM picture on the left bottom. (B) PCR assay of the infected tissues with specific primers for PRRSV. –: Negative control; 1, 2, 3, 4, 5 and 6: the supernatants from the homogenized tissues of lungs, spleen, kidney, heart, liver and brain, respectively. The target fragment of ∼0.6 kb is highlighted by the arrow.

**Figure 5:**
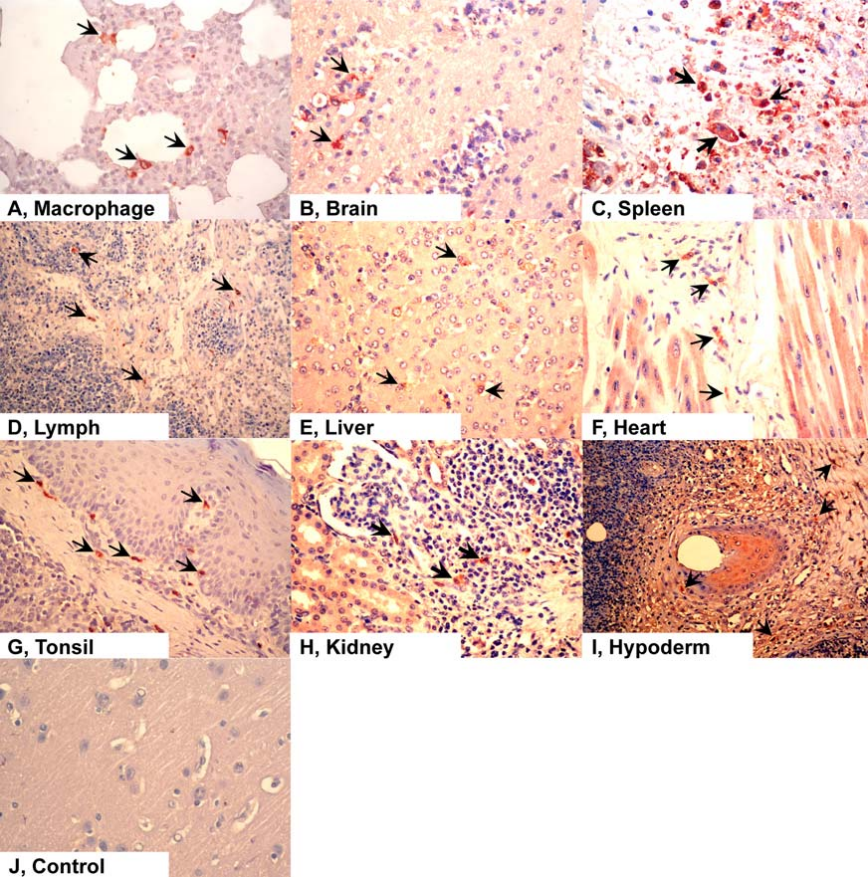
Immunohistochemical analysis of various tissue specimens.

**Figure 6:**
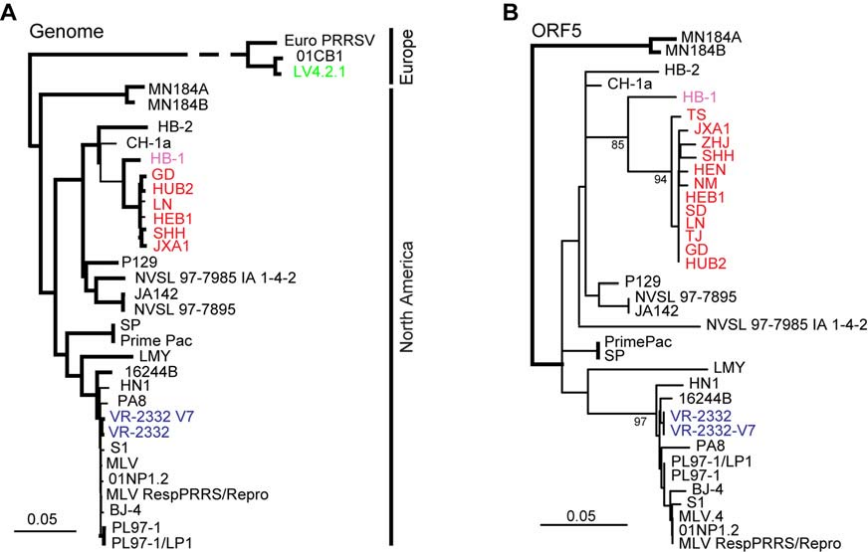
Phylogenetic relationship of PRRSVs. (A) Phylogenetic tree of the isolates in this outbreak together with some other PRRSV strains with known genome sequences. (B) Phylogenetic analysis of PRRSV isolates from this study on the basis of amino acid sequences of ORF5. Both are unrooted trees. PRRSV isolates from this outbreak are highlighted in red; HB-1, a previously reported Chinese isolate is indicated in pink; VR2332, standard strain of Type II (Northern American PRRSV) is shown in blue; and prototype of Type I (European PRRSV) is in green.

**Figure 7:**
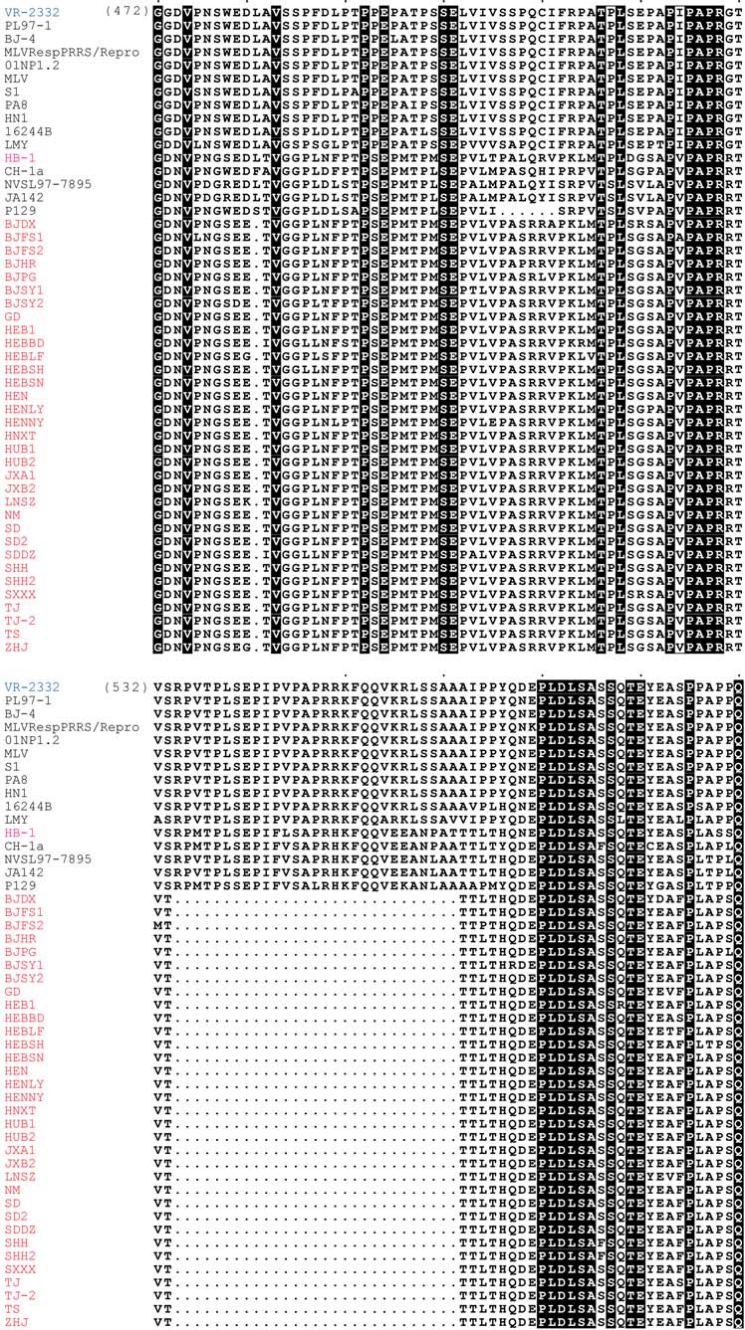
Alignment of NSP2 at the amino acid level. PRRSV isolates from this epidemic are highlighted in red, HB-1, a previously reported Chinese isolate is indicated in pink, and VR2332, the standard strain of Northern American PRRSV is shown in blue.

To gain insights into the pathological changes of those representative death cases, autopsies were first performed on representative death cases. As a result, multiple lesion sites in viscera were found: 1) foci with pathological changes and hyperplasia in lungs together with lung haemorrhagic spots (Fig. 3A) and lung edema (Fig. 3B); 2) spleen infarct and bladder dilatation filled with reddish brown urine (Fig. 3C); 3) frequent blood spots in kidney (Fig. 3D); 4) the putrescence of cardiac muscle (Fig. 3E); 5) foci of the yellow-white necrosis or haemorrhage in liver (Fig. 3F); 6) slightly softened encephala, blood egression emission, and effusion of jelly from the brain putamen (Fig. 3G&H); 7) obvious haemorrhagic spots in lymph node (Fig. 3I); 8) arthritis with swollen joints and intestine ulceration (not shown).

### Identification of the Etiological Agent

Faced with this large-scale outbreak of unknown pathogens, we were contacted by the Ministry of Agriculture in China to promptly form a collaborative investigation team. Our remit was to carry out an extensive and systematic investigation to determine this mysterious causative agent. Initial suspected agents included a variety of pathogens such as pathogenic bacteria (e.g., *Streptococci*), *Mycoplasma*, and viruses (e.g., PRRSV). Therefore, various samples from the dead pigs (listed in Table S1) were used to propagate the possible pathogens in the selection screening media. Subsequently, viral infection was believed to be the most likely cause, although different kinds of pathogens (including *E. coli, Streptococcus, Mycoplasma*, etc.) were found in some samples (not shown). Of note, viruses were always observed in the above cultures by electron microscopy (Fig. 4A). As expected, PCR-based assays showed that many molecular markers for PRRSV could be amplified using the primers specific to the unique gene fragments (e.g., ORF6 3′-NCR) of PRRSV (Fig. 4B). This implies that the highly pathogenic PRRSV infection in pigs may be responsible for the 2006 epidemic of the so-called “high fever” disease in China.

### Immunohistochemical Analysis

To further test the above hypothesis that PRRSV is the causative agent for this outbreak, immunohistochemistry experiments were conducted on paraffin-embedded sections from the autopsy tissue specimens (such as liver, heart, and brain) using a specific anti-PRRSV monoclonal antibody developed by Yang *et al.* [39], [40]. A series of results all showed obvious amaranth/red immunohistochemical staining, indicating the presence of viral antigens (Fig. 5). From the above data, we observed PRSSV infection in all tissues examined, including macrophage (Fig. 5A), brain (Fig. 5B), spleen (Fig. 5C), lymph node (Fig. 5D), liver (Fig. 5E), heart (Fig. 5F), tonsil (Fig. 5G), kidney (Fig. 5H), and hypoderm (Fig. 5I). Therefore, we conclude that the mysterious “high fever” disease might be caused by the highly virulent PRRSV infection.

### High Virulence of the Isolated PRRSV

In order to confirm the above notion, it is essential and important to reproduce the high pathogenicity of the isolated PRRSV by employing a pig infection model. Three representative PRRSV isolates with different origins (JXA1, HEB1, and HUB2) were used for challenge of 12 SPF-pigs (4 piglets/group). In each group, two of the piglets were intravenously injected and both died within 6–8 days, implying the high virulence of the tested PRRSV strains. Similarly, the two other piglets in each group were intranasally inoculated, and they developed marked signs of “high fever” disease (e.g., high fever, blood spots, petechiae, shivering, lamping, etc.) within 3–6 days, and both died on day 10 post-infection. Subsequently, viral isolates were successfully recovered from the infected pigs and confirmed by PCR detection and EM. Autopsies were undertaken to evaluate the immunological effects and pathological lesions. We found almost the same pathological changes (in lung, heart, brain, kidney, liver, etc.) as were observed in pigs killed during the “high fever” epidemic, as well as from the results of our immunohistochemical experiments (Fig. S1). In light of the above data, we are confident that the 2006 outbreak of “high fever” disease in China was caused by highly pathogenic PRRSV infection in pig populations. It is therefore important to understand whether a virulent strain of PRRSV emerged in China, or if novel virulence factors have been acquired by PRRSV ancestors during evolution under some local selection pressures [31], [32], [41].

### Genome-wide Molecular Dissection of the Pathogen

To shed light on the molecular mechanism underlying the high virulence of the isolated PRRSV, six isolates (GD, JXA1, HEB1, SHH, LN and HUB2) were subjected to whole genome sequencing using T vector-based clones. The acquired sequences were assembled into complete genomes with a full length of 15,320 bp, excluding their poly-A sequences. To define their genetic backgrounds, we constructed a phylogenetic tree using their genome sequences together with full genomes obtained from GenBank. Phylogenetic analysis clearly demonstrates that the six strains of PRRSV are extremely similar and can be classified into the same clade. More importantly, they are assigned to Type II with the Northern American strain ATCC VR2332 as a prototype (Fig. 6A). This was also verified by the evolutionary analysis of ORF5, a putative virulence marker at the amino acid level (Fig. 6B). As reported previously [12], HB-1 and HB-2, two Chinese isolates, also belong to Type II PRRSV. The accumulated data reveals that our isolates (e.g., JXA1) share a close relationship with HB-1, suggesting the possibility that PRRSV isolates collected in this outbreak may have evolved from its ancestor.

To search for clues to the high virulence of PRRSV, we continued to perform extensive bioinformatics analysis on all PRRSV genes that encode 6 structural proteins and 13 nonstructural proteins (NSPs) (not shown). Unexpectedly, we identified a unique feature in the NSP2 protein, a potential virulence-associated factor. Among all 33 strains of viruses sequenced in our study, there are two sites where deletion events occurred (Fig. 7). First, a conserved leucine (L) is absent at amino acid position 482. Second, a continuous deletion of 29 aa occurs from amino acid positions 534 to 562. To our knowledge, these unique molecular indicators in NSP2 are only found in our highly pathogenic PRRSV isolates, implying they may possibly serve as novel virulence markers with a pivotal role in the high virulence of PRRSV.

## Discussion

Since its first appearance in the United States and Canada, PRRS has been known to be a serious swine disease associated with major economic losses worldwide [5], [6]. Moreover, typical PRRS is also called “blue ear” disease due to a representative symptom of the infected piglets (i.e., “blue ear”) [42]. Prior to this outbreak of PRRS, we were aware that Northern American (VR2332)-like PRRSVs, which include the HB-1, HB-2, and BJ-4 strains, exist in China [12], [43]. However, there is no evidence to suggest they have ever caused a large scale epidemic of PRRS with fatal cases among grown sows [12], [25], [43]. Here, we describe for the first time an unprecedented outbreak of highly pathogenic PRRS in China in 2006, which spread to more than 10 provinces (autonomous cities or regions) and infected over 2,000,000 pigs, resulting in about 400,000 fatal cases. This epidemic persisted for nearly 3 months and caused considerable economic losses among local pig farmers. Fortunately, thanks to a collaborative effort, the outbreak was finally effectively controlled through the combined means of efficient containment, slaughtering, and vaccine inoculation.

Clinically, this atypical PRRS differs greatly from the general “blue ear” disease, and was characterized by the following symptoms: high fever (40–42°C), petechiae, erythematous blanching rash, etc. Particularly, in these atypical PRRS outbreaks, many adult pigs and some pregnant sows, died as a result of the infection. However, in previous occurrences of PRRSV infection, most of the fatal cases involved young pigs [9], [27], [44]. In addition, both the short disease course of 5–20 days and high contagiousness implied that highly virulent strains of the PRRSV might be involved in this PRRS outbreak, indicating the likelihood of some important mutation of these strains. Autopsy results from dead pigs demonstrated severe damage of multiple organs such as the lungs. Further immunohistochemical analysis also showed positive results, supporting infection of PRRSV in the above organs. Experimental animal infection successfully reproduced the typical symptoms observed in the field. Therefore, we demonstrated that “high fever” disease is essentially caused by PRRSV infection in pig populations, ruling out the possibility of other pathogens.

Comparative genomics analysis revealed that the nucleotide sequences of our PRRSV isolates show a high percentage of similarity to those of a previously reported Chinese strain (HB-1), indicating they belong to the Northern American genotype [12]. Bioinformatics analysis identified a unique molecular feature in NSP2, namely a discontinuous deletion of 30 amino acids (Fig. 7). To our knowledge, this is only present in our strains isolated from the 2006 PRRS outbreak in China, implying its potential role as a virulence marker for the high pathogenicity of PRRSVs, which should be further pursued by developing an infectious clone using the isolated virus [18], [45], [46]. One possible reason, which may help to explain this PRRS epidemic, is that the ancestor PRRSVs with Northern American origin have evolved into highly pathogenic strains under selection pressure in China [31], [41]. Environmental factors such as temperature and relative humidity in the summer [47] and secondary bacterial infections may contribute to the generation of highly virulent PRRS [48], [49].

In conclusion, we have systematically documented an unprecedented large-scale outbreak of mysterious pig disease, named locally as “high fever”, from epidemiology to comparative genomics. The Northern American-like PPRSV virulent strains were found to be the etiological agents for this epidemic. More importantly, a novel molecular hallmark of the PRRSV genomes identified in those pathogens isolated from this outbreak may be correlated with the high pathogenicity, but further experimental validation is required in the near future.

## Methods

### Epidemiological and Clinical Investigation

Following a request by the Ministry of Agriculture in China, a team was organized to perform an epidemiological survey into the large-scale endemic of this so-called “high fever” disease through collaboration with all local veterinary departments [50]. All the statistical data were collected through the local veterinary authorities and standardized in our center. The final data were validated by veterinary authority of the Ministry of Agriculture, therefore the data reported were authoritative. Several representative samples of the infected pigs were collected from different pig farms in different provinces. Briefly, both nasal swabs and different viscera (e.g., brain, heart, liver, spleen, lung, etc) were sampled from dead pigs after autopsy. Subsequently, the samples were placed in sterilized 1×PBS buffer, homogenized, and centrifuged to harvest supernatants as inoculates. The inoculum from each sample was used to propagate the suspected agents responsible for the epidemic. All work on autopsy samples as well as the following immunohistochemistry experiments were conducted by the China Animal Disease Control Center (CADC) in a bio-safety level 3 (BSL-3) facility.

### Determination of the Causative Agent

Initially, the suspicious pathogens varied from the pathogenic bacteria (such as *Streptococcus, E. coli*, and *Staphylococcus*) and *Mycoplasma*, to viruses exemplified by Swine influenza virus (SIV), PCV-2, PRRSV, etc. Thus, a series of microbiological methods were utilized to propagate inocula from the above specimens. They included CHRO Magar ECC media (*E. coli*), blood agar plates (used for selective cultivation of *Pasteurella, Staphylococcus* and *Streptococcus*) [50], Marc-145 cell lines for PRRSV [7], etc. After several rounds of screening and identification, the candidate pathogen was subjected to electron microscopy (EM) to obtain morphological evidence (Protocol S1) [50]. Subsequent PCR-based molecular diagnostics were applied to further confirm the identity of the pathogen.

### PCR-based Assay

To obtain further molecular evidence, the PCR technique was first applied. Briefly, total RNAs were extracted from the viral culture propagated with the inocula of tissue samples using Trizol reagents (Gibico). Routine RT-PCR was carried out according to the standard procedures. Subsequently, 5 sets of specific primers for PRRSV were applied to amplify the following segments: Orf2-1, Orf2-2, Orf2-3, Orf5-7, Orf6 and 3′-NCR that can play roles in the qualitative diagnosis of PRRSV [51].

### Immunohistochemical Experiments

To detect specific antigens in the infected tissues, immunohistochemistry was employed to study a series of formalin-fixed paraffin-embedded specimens that included brain, lung, spleen, liver, heart, lymph node, tonsil, and kidney [50]. The primary antibodies (anti-PRRSV) were produced using the hybridoma cell line, 15A, (kindly provided by Dr. L. Yang from the USA) [39], [40]. The Histo-stain Plus Kit (Zymed Laboratories, South San Francisco, United States) was used for detection of PRRSV. Sections were counterstained with haematoxylin.

### Infection of Experimental Animals

To reproduce what we observed in the field, laboratory infection experiments were performed using 16 SPF-piglets. Three newly-isolated PRRSV strains, termed JXA1, HEB1, and HUB2, were used and they were isolated from dead pigs in Jiangxi Province, Hebei Province, and Hubei Province, respectively. One virulent strain (termed LB2) of PRRSV (collected in our laboratory) was used for positive control. The viral titers of nasal and injection challenge were 1.0 ml and 0.5 ml of virus culture (10^7^ PFU/ml), respectively. The challenged pigs (4 piglets/group) were monitored daily for clinical signs. Viral isolates were recovered from those animals showing apparent clinical symptoms. Autopsies were performed for further examination of the immunological effects and pathological lesions. All procedures were conducted in a BSL-3 facility and approved by the Chinese CADC ethics committee.

### Whole Genome Sequencing of the Isolated PRRSVs

To dissect the possible molecular markers linked with the high virulence of PRRSV responsible for the serious outbreak, six representative viruses were selected for whole-genome sequencing [1], [12], [52]. First, the overlapped cDNA fragments covering the full genome were obtained with universal primers for PRRSVs. Second, the T-vector cloning strategy was employed to generate the recombinant plasmid for direct sequencing with an ABI Sequencer 3730. Third, the acquired sequences were assembled into a complete genome with the aid of Vector NTI Site 9.0 software (Invitrogen). Finally, the six acquired genome sequences (GD, JXA1, HEB1, SHH, LN and HUB2) were deposited in GenBank with accession numbers (EF112445, EF112447, EF112446, and the other three under application)

### Molecular Analysis of the Isolated PRRSVs

To probe the evolutionary relationship between our virus isolates and all other published PRRSVs, a phylogenetic tree was constructed. In brief, the whole genomes (GD, JXA1, HEB1, SHH, LN and HUB2) as well as ORF5 [53], a universal evolutionary scale [22], [23], were aligned using **CLUSTALX 1.83** [54] and then examined manually. Subsequently, we used the neighbor-joining method with 1000 bootstraps on the alignments with the programs **SEQBOOT, DNADIST, PROTDIST, NEIGHBOR,** and **CONSENSE** in the **PHYLIP 3.65** software package. Furthermore, NSP2 (for this gene, we sequenced 33 strains in total) was used to examine the alignment at the amino acid level [18], [24], [46]. In order to clarify that the deletions exist only in the strains we isolated, we did BLAST searches in GenBank with both VR-2332 and HEN sequence segments flanking the deletions as queries [55].

## Supporting Information

Protocol S1 Detailed Materials and Methods
(0.03 MB DOC)

Table S1 Incomplete statistics of representative samples collected from PRRS epidemic sites
(0.09 MB DOC)

Figure S1 Immunological detection of the tissue specimens from pigs experimentally infected by PRRSV
(3.56 MB TIF)
