# Supplementary material for: Emergence of Fatal PRRSV Variants: Unparalleled Outbreaks of Atypical PRRS in China and Molecular Dissection of the Unique Hallmark
Source: PLoS One. 2007 Jun 13;2(6):e526. doi: 10.1371/journal.pone.0000526 (PMC1885284; doi:10.1371/journal.pone.0000526)
Supplement: Protocol S1 — Detailed Materials and Methods (0.03 MB DOC) [file pone.0000526.s001.doc]

**Protocol S1. Detailed Materials and Methods**

***EM Observation of PRRSV Particles*.** To visualize the suspected causative agent, PRRSV, the prepared tissue specimens were subjected to 5 passages of culture in Marc-145 cells. Briefly, the cultures were centrifuged at 5000 rpm for 30 min, and the supernatant was collected and re-centrifuged at 10000 rpm for 40 min. Finally, the diluted deposition was negatively stained with uranyl acetate and observed with a transmission electron microscope (TEM).

In addition, other cell cultures were collected on day 3 post-inoculation with the candidate etiological agent. They were first routinely fixed in 3% glutaraldehyde, then fixed in 1% osmic acid, and embedded in Epon812. Ultra-thin sections (500-800 nm) were processed, stained with uranyl acetate and lead citrate, and observed using EM (Philips). Meanwhile, the cell culture without inoculation of PRRSV served as the negative control.

***Incomplete Statistics of Pigs Affected in the Epidemic.*** To gain the most detailed data of pigs infected in this epidemic of the atypical PRRS, we were authorized by the Ministry of Agriculture in China and allowed to allocate all of the relevant researchers in CADC to various outbreak sites, nearly half of China. Thanks to their fruitful collaborations (field sampling, samples storing, epidemic reporting, and the raw statistics of local pigs affected in different cities or counties), we could collect all of the local/raw data. Finally, the average death rate was the proportion of total dead pigs (piglets & grown pigs) among total pigs affected by the PRRS. Unfortunately, we failed to obtain the detailed data for the dead grown pigs, thus we could not provide the lethal proportion of grown pigs against the total.
