# Supplementary material for: Emergence of Fatal PRRSV Variants: Unparalleled Outbreaks of Atypical PRRS in China and Molecular Dissection of the Unique Hallmark
Source: PLoS One. 2007 Jun 13;2(6):e526. doi: 10.1371/journal.pone.0000526 (PMC1885284; doi:10.1371/journal.pone.0000526)
Supplement: Table S1 — Incomplete statistics of representative samples collected from PRRS epidemic sites (0.09 MB DOC) [file pone.0000526.s002.doc]

**Table S1.** Incomplete statistics of representative samples collected from PRRS epidemic sites

| Outbreak sites | Sampling time | Sample codes | Detailed information of samples |
| --- | --- | --- | --- |
| Jiangxi  Province | 08-23-2006 | JXA1  JXA2  JXB1  JXB2  JXC1 | Brain, heart, lung, liver, spleen, kidney, lymph node, tonsil, bladder, serum, etc. |
|  | 08-24-2006 | JXD1  JXD2 |  |
| Hubei  Province | 08-29-2006 | HBA1  HBA2 | Brain, heart, lung, liver, spleen, kidney, lymph node, tonsil, bladder, serum, etc. |
|  | 08-30-2006 | HBB1 | Brain, heart, lung, liver, spleen, kidney, lymph node, tonsil, bladder, intestine, serum, etc. |
|  |  | HBB2 | Brain, heart, lung, liver, spleen, kidney, lymph node, tonsil, bladder, serum, etc. |
|  |  | HBC1 | Brain, heart, lung, liver, spleen, kidney, lymph node, tonsil, bladder, intestine, serum, etc. |
| Hunan  Province | 09-01-2006 | HNYYA1  HNYYA2  HNYYA11  HNYYB1 | Brain, heart, lung, liver, spleen, kidney, lymph node, tonsil, bladder, serum, etc. |
|  |  |  | Brain, heart, lung, liver, spleen, kidney, lymph node, tonsil, bladder, intestine, serum, etc. |
|  | 09-02-2006 | HNXTA1 | Brain, heart, lung, liver, spleen, kidney, lymph node, tonsil, bladder, serum, etc. |
|  |  | HNXTA2  HNXTA3 | Brain, heart, lung, liver, spleen, kidney, lymph node, bladder, serum, etc. |
|  |  | HNXTA4 | Brain, heart, lung, liver, spleen, kidney, lymph node, tonsil, bladder, serum, etc. |
|  |  | HNXTA5 | Brain, heart, lung, liver, spleen, kidney, lymph node, bladder, serum, etc. |
|  |  | HNXTB1 | Brain, heart, lung, liver, spleen, kidney, lymph node, tonsil, bladder, intestine, serum, etc. |
| Beijing | 09-03-2006 | BJFS | Brain, heart, lung, liver, spleen, kidney, lymph node, tonsil, bladder, serum, etc. |
|  |  | BJDX |  |
|  | 09-16-2006 | BJSY |  |
|  |  | BJCP |  |
|  |  | BJPG |  |
|  | 09-12-2006 | BJHR |  |
|  |  | BJMY |  |
| Hebei  Province | 09-12-2006 | HEBSH | Brain, heart, lung, liver, spleen, kidney, lymph node, tonsil, bladder, serum, etc. |
|  |  | HEBTS |  |
|  | 09-15-2006 | HEBSH |  |
|  |  | HEBZJK |  |
|  | 09-11-2006 | HEBSN | Brain, heart, lung, liver, spleen, kidney, lymph node, tonsil, bladder, intestine, serum, etc. |
|  |  | HEBLF |  |
|  | 09-12-2006 | HEBSJZ |  |
|  |  | HEBXS |  |
|  |  | HEBGBD |  |
| Inner Mongolia | 09-23-2006 | NM | Brain, heart, lung, liver, spleen, kidney, lymph node, serum, etc. |
| Guangdong  Province | 09-03-2006 | GD1 | Brain, heart, lung, liver, spleen, kidney, lymph node, tonsil, intestine, serum, etc. |
|  |  | GD2 |  |
| Tianjin | 09-08-2006 | TJBD1 | Brain, heart, lung, liver, spleen, kidney, lymph node, tonsil, intestine, serum, etc. |
|  | 09-20-2006 | BJBD2 |  |
| Zhejiang  Province | 09-12-2006 | ZHJ | Brain, heart, lung, liver, spleen, kidney, lymph node, serum, etc. |
| Henan  Province | 09-12-2006 | HENNY1 | Brain, heart, lung, liver, spleen, kidney, lymph node, tonsil, serum, etc. |
|  |  | HENNY2 |  |
|  | 09-18-2006 | HENLY1 |  |
|  |  | HENLY1 |  |
|  | 09-01-2006 | HEN1 |  |
| Shandong  Province | 09-12-2006 | SD1 | Brain, heart, lung, liver, spleen, kidney, lymph node, tonsil, serum, etc. |
|  |  | SDZC |  |
|  | 09-10-2006 | SDDZ |  |
|  |  | SDQF |  |
|  |  | SDFC |  |
| Shanghai  Province | 09-12-2006 | SHH1 | Brain, heart, lung, liver, spleen, kidney, lymph node, tonsil, serum, etc. |
|  |  | SHH2 |  |
| Liaoning  Province | 09-12-2006 | LN | Brain, heart, lung, liver, spleen, kidney, lymph node, tonsil, serum, etc. |
|  |  | LN2 |  |
|  | 09-11-2006 | LNSZ |  |
| Shanxi  Province | 09-10-2006 | SXXX | Brain, heart, lung, liver, spleen, kidney, lymph node, tonsil, serum, etc. |

JXA1: Jiangxi A1; JXA2: Jiangxi A2; JXB1: Jiangxi B1; JXB2: Jiangxi B2; JXC1: Jiangxi C1; JXD1: Jiangxi D1; JXD2: Jiangxi D2; HBA1: Hubei A1; HBA2: Hubei A2; HBB1: Hubei B1; HBB2: Hubei B2; HBC1: Hubei C1; HNYYA1: Hunan yiyang A1; HNYYA2: Hunan yiyang A2; HNYYA11: Hunan yiyang A11; HNYYB1: Hunan yiyang B1; HNXTA1: Hunan xiangtan A1; HNXTA2: Hunan xiangtan A2; HNXTA3: Hunan xiangtan A3; HNXTA4: Hunan xiangtan A4; HNXTA5: Hunan xiangtan A5; HNXTB1: Hunan xiangtan B1; BJFS: Beijing fangshan; BJDX: Beijing daxing; BJSY: Beijing shunyi; BJCP: Beijing changping; BJPG: Beijing pinggu; BJHR: Beijing huairou; BJMY: Beijing miyun; HEBSH1: Hebei sanhe1; HEBTS: Hebei tangshan; HEBSH2: Hebei sanhe2; HEBZJK: Hebei zhangjiakou; HEBSN: Hebei suning; HEBLF: Hebei langfang; HEBSJZ: Hebei shijiazhuang; HEBXS: Hebei xushui; HEBGBD: Hebei gaobeidian; NM: Neimenggu; GD1: Guangdong 1; GD2: Guangdong2; TJBD1: Tianjin baodi 1; TJBD2: Tianjin baodi 1; ZHJ: Zhejiang; HENNY1: Henan nanyang 1; HENNY2: Henan nanyang 2; HENLY1: Henan luoyang 1; HENLY2: Henan luoyang 1; HEN1: Henan 1; SD1: Shandong 1; SDZC: Shandong zoucheng; SDDZ: Shandong dezhou; SDQF: Shandong qufu; SDFC: Shandong feicheng; SHH1: Shanghai 1; SHH2: Shanghai 1; LN1: Liaoning 1; LN2: Liaoning 2; LNSZ: Liaoning suizhou; SXXX: Shanxi xixian.
