# Supplementary figures and images for: Emergence of Fatal PRRSV Variants: Unparalleled Outbreaks of Atypical PRRS in China and Molecular Dissection of the Unique Hallmark
Source: PLoS One. 2007 Jun 13;2(6):e526. doi: 10.1371/journal.pone.0000526 (PMC1885284; doi:10.1371/journal.pone.0000526)

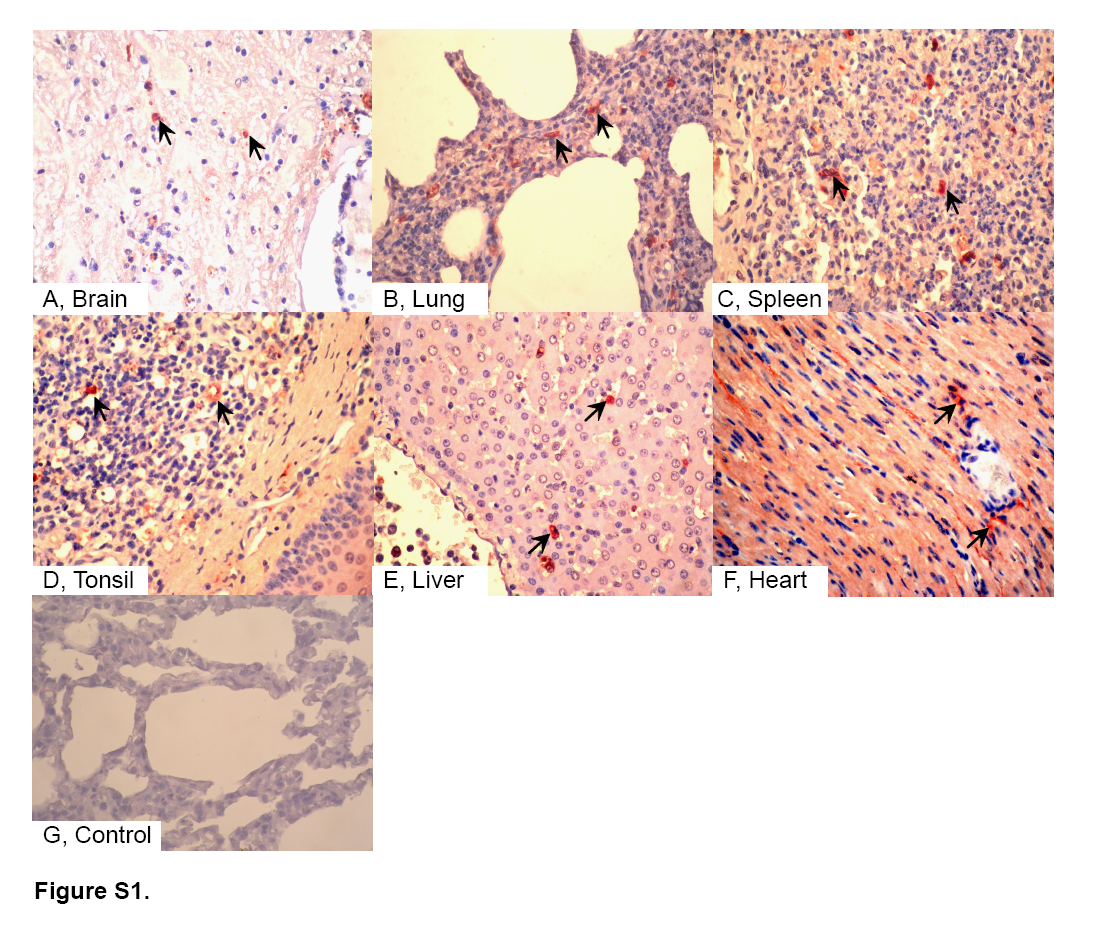

Supplement: Figure S1 — Immunological detection of the tissue specimens from pigs experimentally infected by PRRSV (3.56 MB TIF) [file pone.0000526.s003.tif]
